# Evaluation of critical care burden following traumatic injury from two randomized controlled trials

**DOI:** 10.1038/s41598-023-28422-5

**Published:** 2023-01-20

**Authors:** Insiyah Campwala, Francis X. Guyette, Joshua B. Brown, Mark H. Yazer, Brian J. Daley, Richard S. Miller, Brian G. Harbrecht, Jeffrey A. Claridge, Herbert A. Phelan, Brian Eastridge, Raminder Nirula, Gary A. Vercruysse, Terence O’Keeffe, Bellal Joseph, Matthew D. Neal, Brian S. Zuckerbraun, Jason L. Sperry

**Affiliations:** 1 Division of Trauma and General Surgery, Department of Surgery, University of Pittsburgh, 200 Lothrop St., Pittsburgh, PA 15213 USA; 2 Department of Emergency Medicine, University of Pittsburgh, Pittsburgh, PA USA; 3 The Institute for Transfusion Medicine, University of Pittsburgh, Pittsburgh, PA USA; 4 Department of Surgery, University of Tennessee Health Science Center, Knoxville, TN USA; 5 Department of Surgery, JPS Health Network, Fort Worth, TX USA; 6 Department of Surgery, University of Louisville, Louisville, KY USA; 7 Department of Surgery, Metro Health Medical Center, Case Western Reserve University, Cleveland, OH USA; 8 Department of Surgery, University of Texas Southwestern, Dallas, TX USA; 9 Department of Surgery, University of Texas Health San Antonio, San Antonio, TX USA; 10 Department of Surgery, University of Utah, Salt Lake City, UT USA; 11 Department of Surgery, University of Arizona, Tucson, AZ USA

**Keywords:** Outcomes research, Clinical trial design

## Abstract

Trauma resuscitation practices have continued to improve with new advances targeting prehospital interventions. The critical care burden associated with severely injured patients at risk of hemorrhage has been poorly characterized. We aim to describe the individual and additive effects of multiorgan failure (MOF) and nosocomial infection (NI) on delayed mortality and resource utilization. A secondary analysis of harmonized data from two large prehospital randomized controlled trials (Prehospital Air Medical Plasma (PAMPer) Trial and Study of Tranexamic Acid during Air and Ground Medical Prehospital Transport (STAAMP) Trial) was conducted. Only those patients who survived beyond the first 24 hours post-injury and spent at least one day in the ICU were included. Patients were stratified by development of MOF only, NI only, both, or neither and diagnosis of early (≤ 3 days) versus late MOF (> 3 days). Risk factors of NI and MOF, time course of these ICU complications, associated mortality, and hospital resource utilization were evaluated. Of the 869 patients who were enrolled in PAMPer and STAAMP and who met study criteria, 27.4% developed MOF only (n = 238), 10.9% developed NI only (n = 95), and 15.3% were diagnosed with both MOF and NI (n = 133). Patients developing NI and/or MOF compared to those who had an uncomplicated ICU course had greater injury severity, lower GCS, and greater shock indexes. Early MOF occurred in isolation, while late MOF more often followed NI. MOF was associated with 65% higher independent risk of 30-day mortality when adjusting for cofounders (OR 1.65; 95% CI 1.04–2.6; *p* = 0.03), however NI did not significantly affect odds of mortality. NI was individually associated with longer mechanical ventilation, ICU stay, hospital stay, and rehabilitation requirements, and the addition of MOF further increased the burden of inpatient and post-discharge care. MOF and NI remain common complications for those who survive traumatic injury. MOF is a robust independent predictor of mortality following injury in this cohort, and NI is associated with higher resource utilization. Timing of these ICU complications may reveal differences in pathophysiology and offer targets for continued advancements in treatment.

## Introduction

Traumatic injury is a leading cause of death in the United States and often results in significant long-term disability^1–3^. Trauma resuscitation practices have continued to improve with new advances targeting prehospital interventions, including use of plasma and tranexamic acid^4–9^. However, for those who survive their initial injuries, complications such as nosocomial infection (NI) and multiorgan failure (MOF) remain common and lead to worse outcomes^10–13^.

Recent studies have shown that hemorrhagic shock following severe traumatic injury is a primary risk factor for infection and multiorgan failure in those who survive their injury^1,14,15^. Especially within this cohort of severely injured patients, those who benefit from early prehospital interventions and survive their injury may be more likely to develop these ICU complications^16^.

The critical care burden—or the burden of complications, mortality, and treatment needs—associated with severely injured patients at risk of hemorrhage has been poorly characterized. We aim to describe the individual and additive effects of MOF and NI on patient outcomes, delayed mortality, and resource utilization using data from two large prehospital randomized controlled trials. Characterization of the timing of such complications and their relationship to mortality is essential to improve delayed post-injury outcomes. We hypothesize that in those who survive their initial injury, the development of NI and MOF will be associated with greater risk of mortality and increased resource utilization.

## Methods

We performed a secondary analysis of harmonized data derived from the Prehospital Air Medical Plasma (PAMPer) Trial and Study of Tranexamic Acid during Air and Ground Medical Prehospital Transport (STAAMP) Trial.

The PAMPer Trial (NCT01818427) was a multicenter, prehospital, cluster-randomized trial that compared outcomes in injured patients at risk of hemorrhagic shock who received thawed plasma versus standard-care resuscitation during air medical transport from 2014 through 2017. Patients were eligible for enrollment if they had at least one episode of hypotension (systolic blood pressure < 90 mmHg) and tachycardia (heart rate > 108 beats per minute), or if they had any severe hypotension (systolic blood pressure < 70 mmHg) either before arrival of air medical transport or any time before arrival at the participating trauma center^4^.

The STAAMP Trial (NCT02086500) was a double-blinded, multicenter, prehospital, randomized trial in which differing doses of tranexamic acid (TXA) versus placebo were administered in the prehospital setting to patients at risk of hemorrhage from 2015 to 2019. Patients were eligible for inclusion if they experienced at least 1 episode of hypotension (systolic blood pressure < 90 mmHg) or tachycardia (heart rate > 110 beats per minute) before arrival at the participating center^5^.

Both studies employed exception from informed consent (EFIC) enrollment, after a period of community consultation and public notification. Patients were excluded if they were younger than 18 years of age or older than 90 years of age, intravenous or intraosseous access could not be established, had an isolated fall from standing, had a cervical cord injury, were a known prisoner or were pregnant, had a traumatic cardiac arrest that lasted longer than 5 min, had a penetrating brain injury, had injury due to isolated drowning or hanging, had isolated burns, or were admitted as an inpatient to an outside hospital.

PAMPer (STUDY20070132) and STAAMP trials ((STUDY19060072) were both approved by the University of Pittsburgh Institutional Review Board and at all other study sites. Informed consent was obtained from all subjects enrolled in each of the trials. All study methods were carried out in accordance with relevant guidelines and regulations.

### Study population

Inclusion criteria for this secondary study were patients who were enrolled in the PAMPer trial (501 patients) and the STAAMP trial (903 patients), who survived beyond the first 24 hours post-injury, and who required at least one day of ICU-level care. These criteria were used to eliminate those patients enrolled in either of the trials who were too severely injured and suffered mortality before MOF or NI could develop or who were not severely injured and at lower risk of hemorrhagic shock, MOF, and NI.

### Definitions

Day 0 was considered day of admission. NI was defined as positive culture evidence beyond the day of admission. If patients developed multiple nosocomial infections, the first diagnosed NI was used to calculate time to infection and for descriptive characteristics. Multiorgan failure was defined by the Denver Multiple Organ Dysfunction scoring system. The Denver criteria assigns scores to cardiac, respiratory, renal, and hepatic dysfunction and defines multiple organ failure as two or more organ systems failing (resulting in a score > 3) at least 48 h after significant injury^13,17^. Procedures required in the first 24 hours post-injury were analyzed and were specifically defined as operative interventions performed in the operating room. Resource utilization refers to use of hospital resources, including mechanical ventilators and respiratory support staff, hospital bed, food, nursing support, medication needs, laboratory and imaging needs during an ICU or hospital stay, procedures undergone, and resources needed for safe discharge—including rehabilitation and skilled nursing needs. In this study, resource utilization was measured using parameters such as having had a procedure in the first 24 hours, mechanical ventilation days, ICU length of stay, hospital length of stay, and discharge disposition. Thirty-day outcomes were missing for six patients who were alive at discharge prior to 30 days; for this study, in-hospital mortality was used as a proxy for 30-day mortality for those six patients.

### Statistical analysis

The PAMPer and STAAMP databases were first combined and harmonized. Patients who died within 24 hours of injury and patients who spent less than one day in the intensive care unit were excluded from further analysis. Patients were stratified by development of MOF only, NI only, both MOF and NI, or neither ICU complication. Patient demographics, injury characteristics, and outcomes of patients who developed MOF and/or NI versus those who had an uncomplicated ICU course were compared. Timing of these ICU complications relative to hospital admission and mortality was characterized via Kaplan–Meier survival analyses. Patients who developed MOF were further stratified by diagnosis of early (≤ 3 days) versus late MOF (> 3 days)^13^ to compare time-dependent risk factors and outcomes. Chi-square was used for categorical variables, and independent-samples Mann–Whitney U Tests was used for continuous variables. Binary logistic regression was used to characterize independent mortality risk associated with MOF and NI, after adjusting for covariates, including patient demographics, injury severity, and prehospital trial enrollment. Finally, hospital resource utilization attributable to MOF and NI, including mechanical ventilation days, ICU and hospital length of stay, and post-discharge rehabilitation requirements, was evaluated. A *p*-value of ≤ 0.05 was considered significant for all analyses. Data was stored and analyzed using SPSS Statistics, version 27, for Mac (IBM, Armonk, NY).

### Ethical approval and consent to participate

PAMPer (STUDY20070132) and STAAMP trials ((STUDY19060072) were both approved by the University of Pittsburgh Institutional Review Board and at all other study sites.

### Conference presentation

Presented at Academic Surgical Congress, Orlando, Florida, February 2022.

## Results

**Figure 1:**
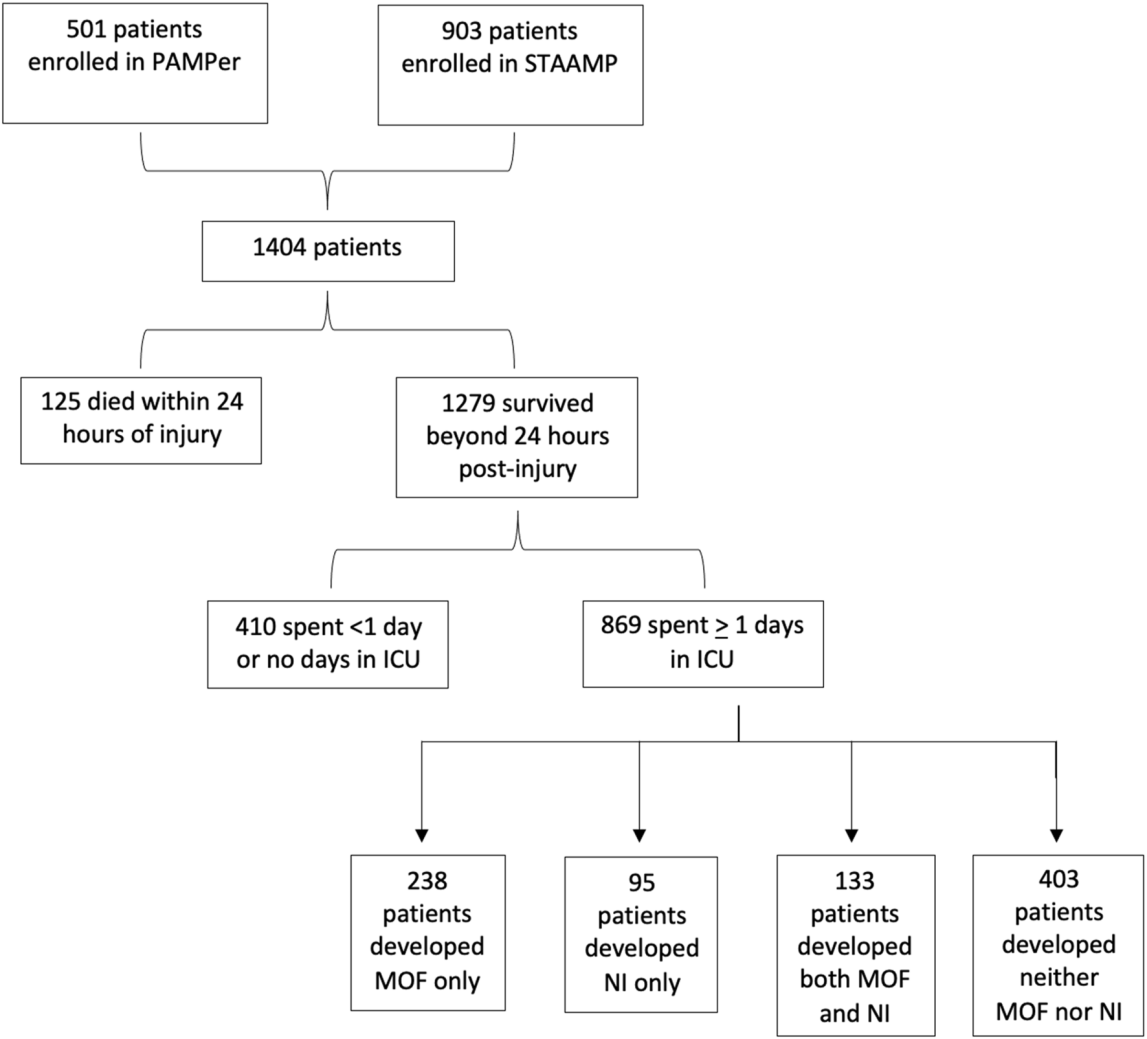
Patient selection flowchart.

Of the 1404 patients who were enrolled collectively in the PAMPer and STAAMP trials, 125 patients died within the first 24 hours following injury and 410 spent less than one day in the intensive care unit, excluding them from further analyses. The remaining 869 patients were primarily male (74.9%), suffered blunt injury (84.9%), and were severely injured—with median injury severity score of 20 (IQR 13–29). Patients had a median GCS score of 13 (IQR 3–15), an elevated shock index of 1.28 (IQR 0.93–1.55), and a 10.9% overall 30-day mortality (n = 95). Of these 869 patients who met study criteria, 27.4% developed MOF only (n = 238), 10.9% developed NI only (n = 95), 15.3% were diagnosed with both MOF and NI (n = 133), and the remaining 46.4% had an uncomplicated ICU course (n = 403) (Fig. 1).

Patients developing NI and/or MOF versus those who had an uncomplicated ICU course were similar in sex and mechanism of injury, but were older, had higher injury severity, lower GCS, and greater hospital resource utilization. Those patients who developed NI and/or MOF also had a higher unadjusted 30-day mortality rate (*p* = 0.02) (Table 1).

**Table 1:** Patient and injury characteristics and outcomes across patients with an uncomplicated ICU course versus MOF and/or NI.

	Uncomplicated ICU Course (N = 403)	MOF and/or NI (N = 466)	*p*-value
Median age (IQR)–years	40 (26–56)	47 (31–60)	** < 0.01**
Male sex—no. (%)	310 (76.9)	341 (73.2)	0.20
Race—no. (%)			**0.03**
White	320 (79.4)	398 (85.4)	
African American	35 (8.7)	32 (6.9)	
Asian	0 (0.0)	2 (0.4)	
Other	3 (0.7)	6 (1.3)	
Not reported	45 (11.2)	28 (6.0)	
Blunt injury—no (%)	343 (85.1)	395 (84.8)	0.64
Prehospital intubation—no (%)	136 (33.7)	231 (49.6)	** < 0.01**
Prehospital CPR—no (%)	7 (1.7)	9 (1.9)	0.83
Prehospital blood—no (%)	58 (14.4)	138 (29.6)	** < 0.01**
Median injury severity score (ISS) (IQR)	16 (9–22)	24 (17–34)	** < 0.01**
Median GCS score (IQR)	14 (8–15)	12 (3–15)	** < 0.01**
Median shock index (IQR)	1.03 (0.85–1.35)	1.44 (1.13–1.69)	** < 0.01**
Traumatic brain injury on CT—no (%)	111 (27.5)	185 (39.8)	** < 0.01**
Procedure in 1st 24 h—no (%)	201 (49.9)	319 (68.5)	** < 0.01**
Median mechanical vent days (IQR)	2 (0–3)	7 (3–13)	** < 0.01**
Median ICU length of stay (IQR)	3 (2–5)	10 (5–17)	** < 0.01**
Median hospital length of stay (IQR)	7 (4–11)	18 (11–28)	** < 0.01**
30-day mortality—no (%)	33 (8.2)	62 (13.3)	**0.02**
Discharge disposition—no (%)			** < 0.01**
Home	208 (51.6)	129 (27.7)	
Rehab	62 (15.4)	97 (20.8)	
Skilled nursing	59 (14.6)	82 (17.6)	
Other	74 (18.4)	158 (33.9)	

Significant values are in bold.

**Figure 2:**
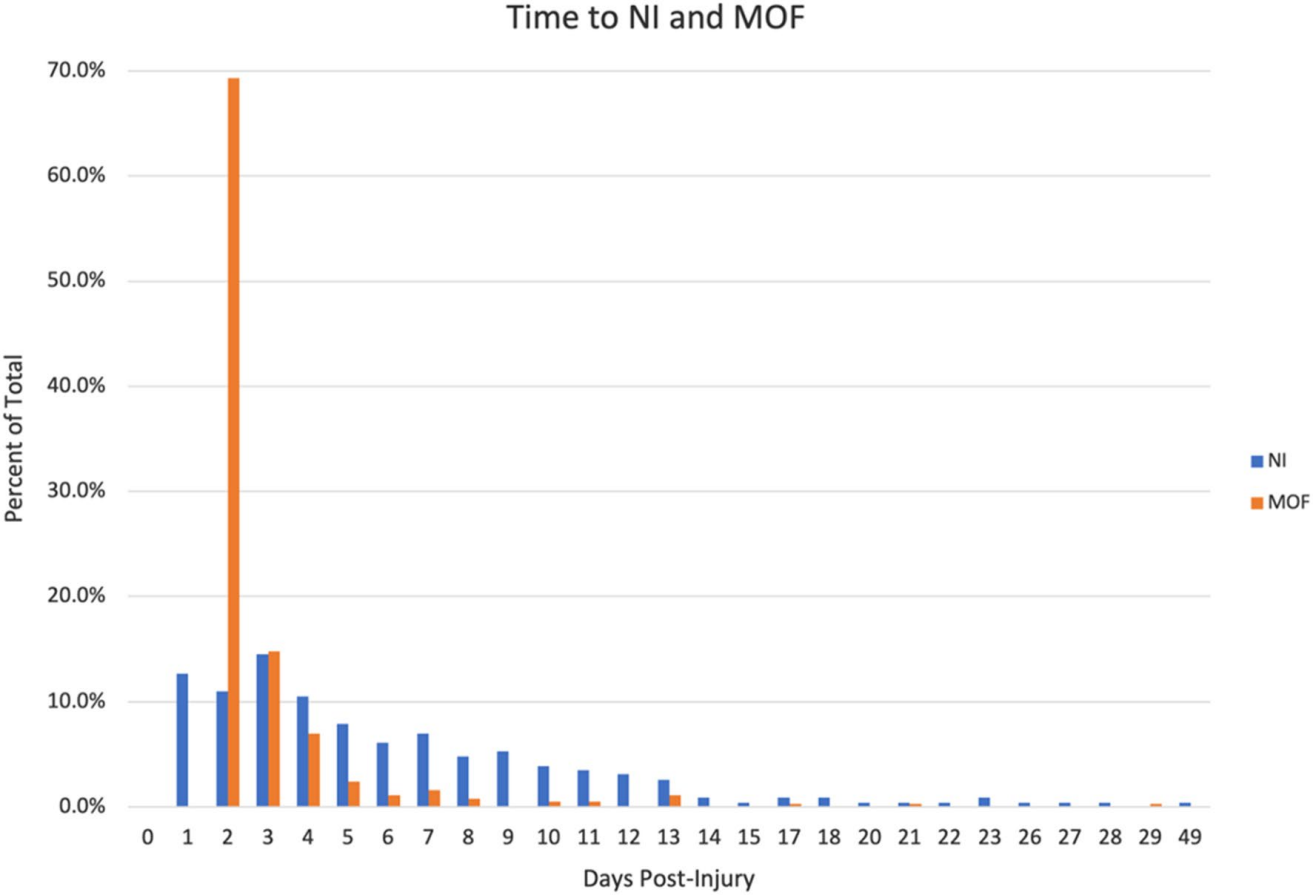
Time to NI and MOF.

Average time to MOF diagnosis was approximately three days (2.9 ± 2.5 days, median 2.0 days (Fig. 2).

Patients who developed MOF versus those who did not were similar in demographics and mechanism of injury but had higher injury and shock severity. They also had a higher unadjusted incidence of 30-day mortality (15.1% vs. 7.8%, *p* < 0.01).

Nosocomial infections were often diagnosed within the first week following injury (mean 6.4 ± 5.8 days, median 5.0 days) (Fig. 2). Those who developed NI versus those who did not were similar in demographics but had greater injury severity and lower GCS. Patients who developed NI did not have a significantly different unadjusted 30-day mortality rate versus those who were not diagnosed with NI (11.8% vs. 10.6%, *p* = 0.61). Most of the 228 patients who developed nosocomial infection post-injury were diagnosed with pneumonia (69.7%), and most infections were from gram-negative organisms (42.1%) (Table 2).

**Table 2:** Infection types.

Infection types	Frequency N (%) N = 228
Pneumonia	159 (69.7%)
Urinary tract infection	25 (11.0%)
Post-op surgical site/intra-abdominal infection	12 (5.3%)
Hematological infection	8 (3.5%)
Other	24 (10.5%)

*Other: fungal infections, tibial infection, pleural cavity infection/empyema, Clostridioides difficile diarrhea and colitis, soft tissue infection, corneal infection.

While 161 patients developed only one nosocomial infection post-injury, 44 patients developed two infections, 18 patients developed 3 infections, 2 patients developed 4 infections, and 3 patients developed 5 infections. Those 67 patients who developed two or more infections during their admission did not have a significantly different incidence of unadjusted 30-day mortality versus those with one infection (7.5% versus 13.7%, *p* = 0.19).

### Timing of ICU complications

The incidence of NI was 38.2% (n = 87) in the first three days and reduced over time over the initial 30 days, while the majority of MOF was diagnosed within the first three days post-injury (n = 312, 84.1%) (Fig. 2). Patients developing late MOF were similar in age, sex, mechanism of injury, and shock index compared to patients developing early MOF but had higher injury severity and greater incidence of concomitant NI. Early MOF (≤ 3 days) was more likely associated with diagnosis of only MOF (66.3% vs 52.5%), while late MOF (> 3 days) was more likely associated with diagnosis of both MOF and NI (47.5% vs 33.7%, *p* = 0.04). Of those patients who developed both MOF and NI, early MOF more often preceded NI, while late MOF more often temporally followed the diagnosis of NI (*p* < 0.01).

### Mortality

**Figure 3:**
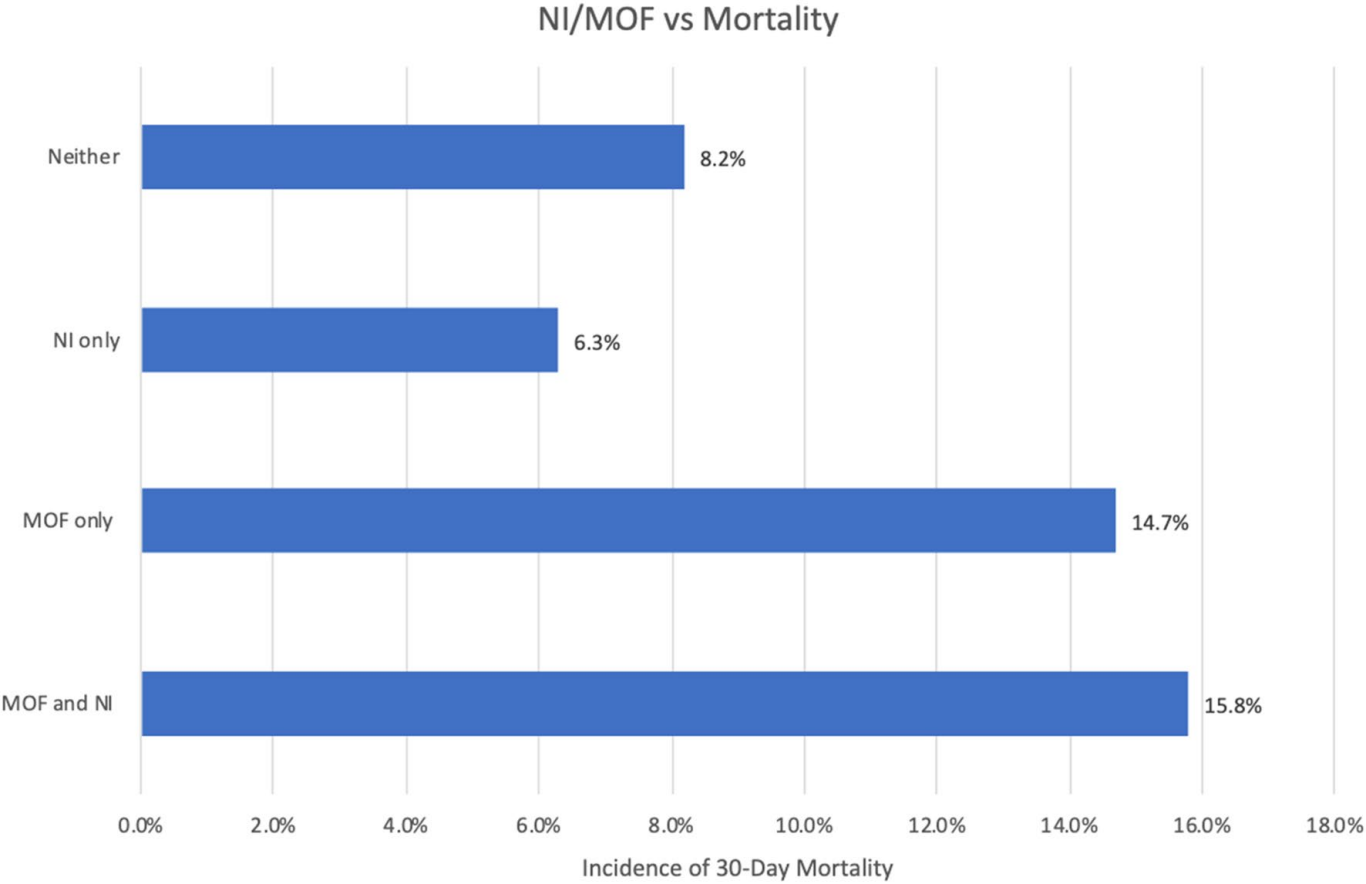
Unadjusted mortality associated with NI and MOF.

Patients who developed only NI had a 6.3% incidence of 30-day mortality; however, isolated MOF and the combined MOF and NI group were associated with higher mortality (14.7% and 15.8%) (Fig. 3).

When adjusting for age, gender, injury severity, and prehospital trial enrollment, multiorgan failure was associated with 65% higher independent risk of 30-day mortality (OR 1.65; 95% CI 1.04–2.6; *p* = 0.03); however, nosocomial infection did not significantly affect odds of 30-day mortality (OR 0.83; CI 0.51–1.36; *p* = 0.46) (Table 3).

**Table 3:** Predictive factors for 30-day mortality.

Predictors	Odds Ratio	(95% CI)	*P*-value
Age	1.03	1.01–1.04	** < 0.01**
Gender (M/F)	0.69	0.40–1.17	0.17
ISS > 15	2.62	1.45–4.74	** < 0.01**
Enrollment in PAMPer (vs STAAMP)	0.98	0.61–1.58	0.94
NI	0.83	0.51–1.36	0.46
MOF	1.65	1.04–2.60	**0.03**
Constant	0.02		** < 0.01**

OR: Odds Ratio, CI: Confidence Interval, ISS: injury severity score; NI: nosocomial infection; MOF: multiorgan failure.

Significant values are in bold.

**Figure 4:**
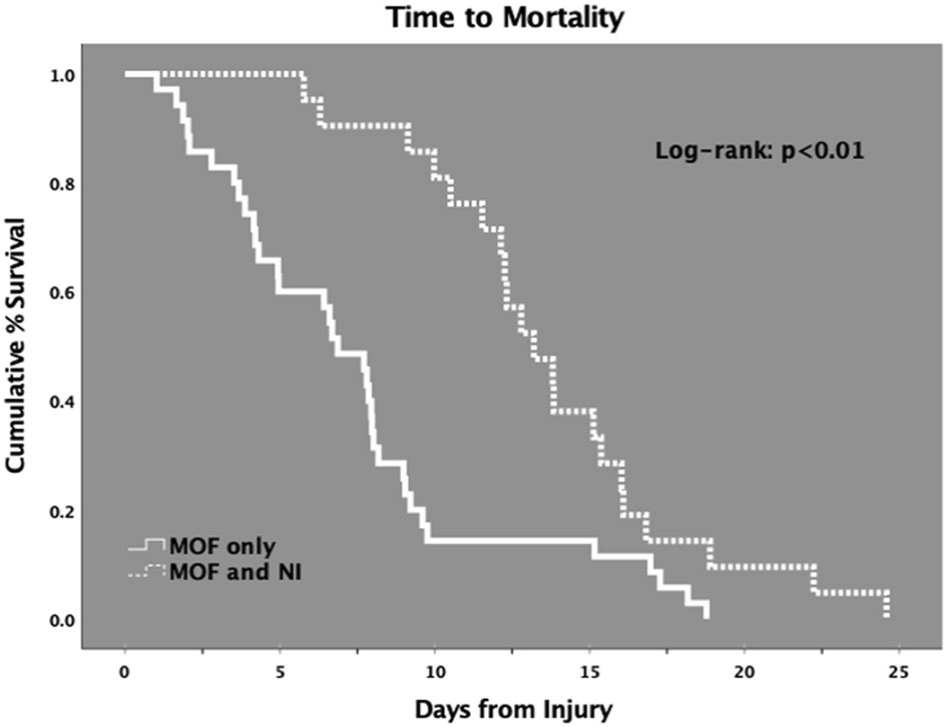
Time to mortality.

There was no significant difference in 30-day mortality between early versus late MOF (14.4% vs 18.6%, *p* = 0.41) and no difference in median time to 30-day mortality between early and late MOF groups (8.19 [IQR 4.26, 12.53] days versus 9.98 [IQR 7.87, 16.83] days; *p* = 0.14). Although there was no unadjusted mortality difference between MOF only versus MOF and NI, among those 56 patients who developed MOF only or MOF and NI and who suffered death by 30 days post-injury, median time to mortality was significantly earlier for those with MOF only (6.88 [IQR 3.88, 9.05] days) versus those who developed both MOF and NI (13.20 [IQR 11.02, 16.06] days) (*p* < 0.01) (Fig. 4).

### Resource utilization

Patients who developed NI and/or MOF following injury more often required an operative intervention within the first 24 h post-injury and had significantly longer periods of mechanical ventilation, ICU stay, hospital stay, and rehab requirements when compared to patients with an uncomplicated ICU course (Table 1). NI was individually associated with longer mechanical ventilation, ICU stay, hospital stay, and rehabilitation requirements, and the addition of MOF further increased the burden of inpatient and post-discharge care. Patients developing two or more nosocomial infections had greater mechanical ventilation needs, longer ICU stays, and longer hospital stays. This suggests that nosocomial infection is the principal driver of resource utilization and the combination of NI and MOF was additive. (Tables 4, 5, 6).

**Table 4:** Hospital resource utilization individually attributable to MOF versus NI.

	NI only (N = 95)	Both MOF and NI (N = 133)	*p*-value
Procedure in 1st 24 h—no (%)	62 (65.3)	95 (71.4)	0.32
Median mechanical vent days (IQR)	6 (3–11)	13 (9–20)	** < 0.01**
Median ICU length of stay (IQR)	9 (5–17)	17 (13–24)	** < 0.01**
Median hospital length of stay (IQR)	19 (12–27)	26 (18–30)	** < 0.01**
30-day mortality—no (%)	6 (6.3)	**21 (15.8)**	**0.03**
Discharge Disposition—no (%)			**0.02**
Home	23 (24.2)	20 (15.0)	
Rehab	27 (28.4)	34 (25.6)	
Skilled nursing	18 (18.9)	16 (12.0)	
Other	27 (28.4)	63 (47.4)	

Significant values are in bold.

**Table 5:** Additive hospital resource utilization of MOF and NI.

	MOF only (N = 238)	NI only (N = 95)	*p*-value
Procedure in 1st 24 h—no (%)	162 (68.1)	62 (65.3)	0.62
Median mechanical vent days (IQR)	4 (2–10)	**6 (3–11)**	**0.03**
Median ICU length of stay (IQR)	8 (4–12)	**9 (5–17)**	**0.02**
Median hospital length of stay (IQR)	15 (8–23)	**19 (12–27)**	** < 0.01**
30-day mortality—no (%)	**35 (14.7)**	6 (6.3)	**0.04**
Discharge Disposition—no (%)			**0.03**
Home	86 (36.1)	23 (24.2)	
Rehab	36 (15.1)	27 (28.4)	
Skilled nursing	48 (20.2)	18 (18.9)	
Other	68 (28.6)	27 (28.4)	

Significant values are in bold.

**Table 6:** Hospital resource utilization of one versus multiple NI.

	1 NI (N = 161)	≥ 2 NI (N = 67)	*p*-value
Procedure in 1st 24 h—no (%)	111 (68.9)	46 (68.7)	0.97
Median mechanical vent days (IQR)	9 (4–15)	14 (9–21)	** < 0.01**
Median ICU length of stay (IQR)	13 (6–19)	19 (14–25)	** < 0.01**
Median hospital length of stay (IQR)	20 (13–30)	28 (21–30)	** < 0.01**
30-day mortality—no (%)	22 (13.7)	5 (7.5)	0.19
Discharge disposition—no (%)			0.20
Home	36 (22.4)	7 (10.4)	
Rehab	40 (24.8)	21 (31.3)	
Skilled nursing	24 (14.9)	10 (14.9)	
Other	61 (37.9)	29 (43.3)	

Significant values are in bold.

## Discussion

The management of the injured patient has evolved over the last decade and recent trials have demonstrated improved outcomes due to changes in early resuscitation practices. As these practices are incorporated and outcome improvements are realized, post-injury complications including NI and MOF may become more common, and the burden associated with these ICU complications remains poorly characterized^16^. The primary analyses of the PAMPer and STAAMP trials did evaluate the incidence of MOF and NI within their study population, and both found that their prehospital interventions did not significantly change incidence of MOF or NI compared to control groups. However, this secondary analysis combined both study populations, selects for the most severely injured patients, excludes early mortality, and evaluates the incidence, risk factors, timing, and resource utilization of MOF and NI, both individually and in combination, which to our knowledge, has not previously been reported. Additionally, while organ failure has been well characterized as an outcome following sepsis, it has been less characterized following injury, particularly as trauma care has continued to improve with changes in trauma resuscitation. In combining two large study populations of severely injured patients at risk of hemorrhage, we were able to evaluate the incidence of these ICU-based complications over a recent time period and determine their associations with subsequent mortality.

In the current study cohort, the incidence of MOF was 42.7%, and the incidence of NI was 26.2%, demonstrating that these outcomes remain common complications in this study population of patients who survived beyond 24 hours following traumatic injury and required at least one day of ICU care. The development of MOF was associated with greater mortality, and NI was associated with greater hospital resource utilization, irrespective of when these events occurred. Early MOF occurred in isolation, while later MOF was commonly preceded by NI.

The current results corroborate prior literature showing that even after excluding early deaths, these ICU complications occur commonly following injury, are associated with high morbidity and mortality, and are resource intensive^10–13,15,18–27^. The current data further examines these complications and shows that MOF is a strong independent risk factor for later mortality, resulting in a 65% increased odds of 30-day mortality after adjusting for important cofounders, while NI was not independently associated with mortality. Within our study population of severely injured patients, 15.1% of patients who developed MOF (both early and late) and 11.8% of patients who developed NI died within 30 days of injury. Prior studies have demonstrated that MOF increases hospital resource utilization and is associated with prolonged disability, but we additionally found that nosocomial infection is the larger contributor to hospital resource utilization when these events are compared individually^12,28^. In our study population, we found that NI both independently and in combination with MOF required longer periods of mechanical ventilation, ICU care, hospital stay, and greater rehabilitation requirements post-discharge. The critical care burden was further increased in patients who developed more than one nosocomial infection during their hospitalization.

Temporal patterns in the incidence of these ICU complications and subsequent mortality were additionally explored. Early MOF, occurring within three days post-injury, more often occurred in isolation, whereas late MOF more likely followed development of NI, supporting a distinction between trauma-induced MOF versus infection-induced organ dysfunction. No significant difference was identified in incidence or timing of 30-day mortality between patients developing early versus late MOF. Post-injury MOF has been previously described as a bimodal phenomenon, and these current results illustrate a similar temporal relationship between early and late MOF^11,13,17^.

It may be that the ICU management of NI has improved over time, which may be why its association with mortality is not seen in the current results^29–31^. However, progress in treatment practices for post-injury MOF may be more difficult and requires continued investigation to ultimately improve these outcomes^11,12^. In the current analyses, mortality attributable to NI occurred later in the ICU course as compared to mortality associated with isolated MOF. Development of NI resulted in significantly greater resource utilization. It may be that a survival bias plays a role, and patients needed to survive in the ICU long enough to have their NI diagnosed, resulting in these associations. Those patients who develop NI were also found to have greater injury severity and lower GCS than those who did not develop NI, so the greater severity of their original injuries may have required longer ICU stays and ventilator days, providing time for development of nosocomial infection. Therefore, the greater resource utilization attributed to infection may be confounded by injury severity.

## Limitations

There are limitations to this study. Our cohort was based upon two randomized trials that had specific inclusion criteria and involved only large academic level 1 trauma centers; therefore, these results may not be generalizable to the general trauma population. Site-specific ICU complications, mortality, and associations could not be assessed using the current trial data. In our secondary analysis, we excluded patients who died within the first 24 hours post-injury, as they would less commonly have time to develop these ICU complications, especially given that the Denver Multiple Organ Dysfunction scoring system defines MOF as two or more organ systems failing at least 48 hours after significant injury. We selected for those patients who required at least one day of ICU-level care, additionally narrowing our cohort to those most severely injured patients.

Our mortality models are robust, but the two studies were harmonized post hoc, and there were important differences between the primary study cohorts, interventions, and outcomes. To mitigate this limitation, our models controlled for patient demographics, injury severity, and trial enrollment. Importantly, prehospital interventions were not shown to significantly alter the incidence of MOF and NI in the primary studies. The definition and timing of NI in the current study was based upon timing of culture evidence, not the time of infection development, which is difficult to characterize. Additionally, we were unable to characterize the severity of infection or incidence of sepsis or septic shock using data provided from these trials; inclusion of a large range of NI may be why infection was not found to be associated with mortality.

The current study is unable to determine if an event that temporally precedes another is causally related. We were also unable to evaluate the number and timing of operative procedures patients underwent beyond the first 24 hours post-injury using data provided from the current trials. Increased resource utilization associated with NI may be confounded by possible prolonged survival and hospital stay relative to those who develop MOF. We did not perform any economic analyses as that data was not available from the current trials.

## Conclusions

With advances in trauma care and early resuscitation practices, more severely injured patients are surviving traumatic injury, and ICU complications, such as MOF and NI, may become increasingly common and require robust critical care support. The current results demonstrate that multiple organ failure is a principal driver of mortality following injury in this cohort, but nosocomial infection contributes to higher resource utilization. The time course over which these ICU complications occur may be important in distinguishing their causal etiologies. As management of traumatically injured patients continues to improve, greater focus on these delayed complications and the management of such may be required. Future studies to validate the current results and continued investigation into improving these detrimental outcomes are needed.

## Data Availability

Following publication of the primary and all secondary analyses detailed in study protocols, individual de-identified data will be available upon request and approval of the proposed use of the data after 3 years of the close of the trial. The trial protocol, statistical analysis plan embedded in the protocol and the trial publications are available on-line. Requests should be sent to the corresponding author.

## Abbreviations

PAMPer: Prehospital Air Medical Plasma Trial
STAAMP: Study of Tranexamic Acid during Air and Ground Medical Prehospital Transport Trial
ICU: Intensive care unit
MOF: Multiorgan failure
NI: Nosocomial infection
IQR: Interquartile range
ISS: Injury severity score
GCS: Glasgow Coma Score
OR: Odds Ratio
CI: Confidence Interval
